# Food Insecurity and Binge Eating: Exploring Reward-Based Eating, Psychological Distress, and Diet Quality as Underlying Mechanisms

**DOI:** 10.3390/nu18132126

**Published:** 2026-07-01

**Authors:** Chelsea Arnold, Courtney P. McLean, Luiza Bonfim Pacheco, Antonio Verdejo-Garcia

**Affiliations:** School of Psychological Sciences and Turner Institute for Brain and Mental Health, Monash University, Level 5, 18 Innovation Walk, Clayton, VIC 3800, Australia; courtney.mclean@monash.edu (C.P.M.); luiza.bonfimpacheco@monash.edu (L.B.P.); antonio.verdejo@monash.edu (A.V.-G.)

**Keywords:** binge eating, food insecurity, reward-based eating, psychological distress

## Abstract

**Background:** Food insecurity is a risk factor for binge eating behaviours and related disorders; however, the mechanisms underlying this association remain unclear. This study examined reward-based eating, psychological distress, and diet quality as potential mechanisms underscoring this association. **Methods:** Participants (*N* = 176) completed self-reports measuring reward-based eating (Reward-Based Eating Drive scale), distress (Depression, Anxiety and Stress scale), and diet (Australian Eating Survey), alongside a well-validated food insecurity survey, as part of a broader biopsychosocial assessment study. Parallel mediation analysis was utilised to explore the candidate variables as potential mediators between food insecurity and binge eating indicated by the Binge Eating Scale. **Results:** Food insecurity independently predicted binge eating behaviours, in association with the mediating variables. Reward-based eating and psychological distress were statistically significant mediators, but diet quality was not. **Conclusions:** Food insecurity is associated with binge eating through both reward drive and distress. Future research should investigate how both social and psychological interventions may leverage these mechanisms to overcoming binge eating.

## 1. Introduction

Food insecurity is the limited or uncertain access to nutritionally adequate and safe foods, or to foods in socially acceptable ways [1]. Food insecurity exists on a continuum, with the food security status of a household ranging from high, characterised by dependable access to adequate and safe foods, to severe, characterised by one or more members of the household having missed a meal and/or reduced food intake [2]. Food insecurity is a global health problem that impacts the health and wellbeing of both resource-poor and resource-rich environments. For example, in Australia in 2023, the prevalence of food insecurity was 13.2% or 1.3 million households [3], and globally, it is estimated to acutely impact 318 million people in 2026 [4]. Food insecurity can also be understood as a critical social determinant of health, with studies identifying contributing factors such as poverty, unemployment, immigration status, disability, education level, health education, geographic location, and age and gender [5]. As a result, food insecurity is linked to numerous poor health outcomes.

There is growing evidence linking food insecurity and binge eating (defined as eating an unusually large amount of food with feelings of loss of control over eating) [1,6,7]. While this association has been well-established in the literature [7], driving mechanisms are not well-understood. Several theories may be relevant in elucidating this association. One predominant hypothesis considers the episodic nature of food insecurity, where food intake decreases when supply is low and increases when food is available, leading to a resource-imposed cycle of restriction. Insufficient access to food may further contribute to a sense of deprivation, and accordingly increase preoccupation with food [8]. This may be followed by a sense of being out of control, when food later becomes available [9]. Accordingly, individuals with food insecurity who experience binge eating may seize opportunities to eat desirable foods when available, noting a motivational and hedonic component of consumption [9]. As such, this externally imposed cycle of restriction may result in increased reward-based eating (i.e., reward sensitivity to highly palatable foods) [7]. Defined as eating driven by reward circuitry rather than energy needs [10], reward-based eating sensitivity may become amplified during food scarcity, further increasing vulnerability to binge eating.

Complementarily, according to affect regulation theory, binge eating occurs as a coping mechanism in attempts to manage negative emotional experiences [11]. Individuals experiencing food insecurity may encounter heightened psychological distress on the background of financial uncertainty, as well as the stress caused by the unpredictability of maintaining or acquiring sufficient food [12,13,14]. Accordingly, greater food insecurity has been associated with increased psychological distress, as well as negative emotional experiences such as stress, anxiety, and depression [13,14,15]. Qualitative reports from individuals with binge eating and food insecurity support this model, highlighting that distress relating to uncertain access to food can lead to episodes of binge eating [16]. Stress has been found to play a key role in explaining the association between food insecurity and emotional overeating amongst a sample of women [17]. Thus, there is support for further investigation of negative emotional experiences as a key mediator between food insecurity and binge eating.

Finally, the relative affordability of energy-dense, palatable foods, typically consumed during episodes of binge eating, could be relevant in explaining the association with food insecurity [18]. The relationship between food insecurity and poorer diet quality has been demonstrated across marginally, moderately, and severely food-secure adults [19]. Food-insecure households are also more likely to live in areas with fewer supermarkets and a greater concentration of fast-food stores [20,21], and have less access to healthier food alternatives. Additionally, households with low incomes, such as those with greater levels of food insecurity, may prioritise cheaper foods, which tend to be higher-calorie and palatable foods [16]. These affordable and appealing processed food options are more likely to be consumed during episodes of binge eating (as opposed to more expensive, nutritious food alternatives), such that the availability of these foods may make binge eating more likely amongst this group [16,18].

Food insecurity may heighten reward sensitivity and psychological distress, and limit access to nutritious foods, leading to greater vulnerability to binge eating. The aim of this study was therefore to investigate psychological distress, reward-based eating, and diet quality as potential mechanisms underlying the association between food insecurity and binge eating. It was hypothesised that food insecurity would be positively associated with binge eating and this association would be mediated by the three variables of interest. Investigating these mechanisms may help inform more targeted interventions that address both structural barriers to food access and individual-level drivers of eating behaviour.

## 2. Materials and Methods

### 2.1. Study Design

The current study utilised data from 176 participants collected as part of the FoodCode study, a cross-sectional case–control investigation into biopsychosocial drivers of food choices; detailed methods are in [22]. The study was conducted in accord with the Declaration of Helsinki and approved by the Monash University Human Research Ethics Committee (MUHREC), reference 34518 (13 September 2022).

### 2.2. Participants

Recruitment for the parent study comprised three groups sampled according to body mass index (BMI) and eating disorder and mental health status: (1) lower-weight healthy individuals (BMI 18.5–25) with no history of eating disorders (*n *= 63), (2) higher-weight individuals (BMI > 30) with no history of eating disorders (*n *= 56), and (3) higher-weight individuals (BMI > 30) meeting criteria for binge eating disorder or other specified feeding or eating disorder with binge eating subtype (*n *= 57) as indicated by the Structured Clinical Interview for DSM 5 [SCID] [23].

Inclusion criteria required all participants to be aged 18 to 60, able to attend an in-person visit at the study setting, and have no history of psychosis, bipolar disorder or anorexia nervosa, or current suicidality (as indicated by the SCID), neurological disorders, or intellectual disability (indicated by self-report). Participants were also excluded from the lower-weight group with no history of eating disorders if they met criteria for a current major depressive episode on the SCID, as we wanted this group to be representative of healthy controls in terms of both weight and mental health.

### 2.3. Measures

#### 2.3.1. Predicting Variable: Food Insecurity

##### U.S. Household Food Security Survey Module: Six-Item Short Form (HFSSM-6)

The U.S. Household Food Security Survey Module: Six-Item Short Form (HFSSM-6 [24]) assesses household food access and security over the past 12 months, focusing on experiences such as worrying about running out of food, reducing meal sizes, or skipping meals due to limited resources. This short form includes six questions (e.g., “*In the last 12 months, did you ever cut the size of your meals or skip meals because there wasn’t enough money for food?*”), which respondents answer based on their household’s experiences. Responses are typically yes/no or frequency-based, and each affirmative answer is scored (possible range 0–6). In the current study, the total score was used, reflecting the overall level of food insecurity, with higher scores indicating greater household food insecurity. Internal consistency was adequate (Cronbach’s α = 0.78).

#### 2.3.2. Proposed Mediators: Reward Drive, Distress and Diet Quality

##### Reward-Based Eating Drive (RED-13)

The Reward-Based Eating Drive (RED-13) scale [25] evaluates three domains of reward-related eating: preoccupation with food, lack of control over eating, and lack of satiety. The scale contains 13 items (e.g., “*When I start eating, I just can’t seem to stop*”), with responses rated on a five-point Likert scale from 0 (strongly disagree) to 4 (strongly agree). In the current study, the total score was used, with higher scores representing a greater overall tendency for reward-based eating (possible range 0–52). Internal consistency was excellent (Cronbach’s α = 0.92).

##### Depression, Anxiety, and Stress Scales (DASS-21)

The Depression, Anxiety, and Stress Scales (DASS-21) [26,27] measures symptoms of depression, anxiety, and stress. This instrument consists of 21 items (e.g., “*I felt that I had nothing to look forward to*”), which participants rate according to how much each statement applied to them over the past seven days. Responses are recorded on a four-point Likert scale ranging from 0 (did not apply to me at all) to 3 (applied to me very much or most of the time). The total score was used in the current study as an index of psychological distress (possible range: 0–63), with higher scores indicative of greater distress. Internal consistency was excellent (Cronbach’s α = 0.92).

##### Australian Eating Survey (AES)

The Australian Eating Survey (AES) [28] evaluates habitual dietary intake and overall diet quality in Australian populations. It includes a food frequency questionnaire that asks participants how often they consume a variety of foods over the past 3–6 months. For example, “*How many times a week do you eat vegetables with your meal at night?*” All items are used to calculate an Australian Recommended Food Score (ARFS) total score, which reflects adherence to national dietary guidelines and overall diet quality. The ARFS was used in this study as a proxy of overall diet quality, with higher scores indicating higher diet quality (possible range 0–73).

#### 2.3.3. Outcome: Binge Eating Behaviour

##### Binge Eating Scale (BES)

The Binge Eating Scale (BES) [29] measures the frequency and severity of binge eating behaviours, and related cognition and emotions. The scale includes 16 items, each offering either three response options (for 2 items) or four response options (for 14 items). Participants select the statement that best describes their experience (e.g., “*I don’t have any difficulty eating slowly in the proper manner*”, “*At times, I tend to eat quickly and then feel uncomfortably full afterwards*”). In the current study, the total score was used, with higher scores indicating greater severity of binge eating (possible range 0–46). Internal consistency was excellent (Cronbach’s α = 0.92).

### 2.4. Procedure

Recruitment was conducted in community settings through email invitations to internal databases, social media advertisements via Meta, and a research recruitment agency, TrialFacts, between 2023 and 2025. Participants expressed their interest by providing informed consent and completed an initial eligibility screening questionnaire online (including the RED-13 and BES).

Participants who met preliminary inclusion criteria on the screening questionnaire were invited to attend a semi-structured clinical interview (Structured Clinical Interview for DSM-5 research version; SCID-V-RV) conducted via Zoom with a member of the research team. During the interview, participants’ eligibility was confirmed, and eligible participants invited to attend an in-person session at the Turner Clinics in Notting Hill, VIC, Australia.

The in-person session was conducted in the morning and lasted approximately 3.5 h. Participants arrived in a fasted state for the collection of blood samples and body composition. Following these measurements, a standardised breakfast was provided. After breakfast, participants completed a battery of cognitive tasks and questionnaires (including the HFSSM-6, DASS-21, and AES used in this study). Participants received an $80 electronic gift card as reimbursement for their participation.

### 2.5. Statistical Analysis

Statistical analyses were performed using IBM Statistical Package for the Social Sciences (SPSS V29, 2022). Descriptive statistics were calculated and bivariate correlations were used to examine the associations between variables. A parallel mediation analysis was conducted using the Hayes’ PROCESS Macro Model 4 (V5) to examine whether reward-based eating (RED-13 total score), distress (DASS-21 total score), or diet quality (ARFS total score) mediated the relationship between food security (HFSSM-6 total score) and binge eating symptoms (BES total score). When determining which variables to adjust for as potential confounders, one important consideration given the study design was whether to include recruitment group status or BMI as adjustment variables. Given that BMI was used in the definition of the recruitment groups, this variable is collinear with group status. To help determine which variables to adjust for, we outline our causal model for how the variables are related in a directed acyclic graph (DAG) in the Supplementary Material (Figure S1), which allows determination of which variables to adjust for based on ensuring that no backdoor paths from exposure to outcome are left open [30,31]. Based on our causal model, age, gender, and years of education were included as covariates because they form common causes of the exposure, mediators and outcome. By contrast, in our model, BMI and participant group status (i.e., eating disorder diagnosis) are caused by the exposure, mediators, and outcome. We base this causal relationship on several reasons. First, eating disorder diagnosis is derived from survey-type items that comprise items assessing the mediators and the exposure and outcome. As such, we reasonably argue that, in our study, the diagnosis arises from these underlying symptoms and is thus caused by them. Moreover, we argue that BMI is caused by the exposure (food insecurity) rather than the converse, as financial strain results in cheaper foods of lower nutritional quality (i.e., the mediator variable of diet quality) which are more calorically dense and contribute to higher weight. We additionally propose that reward-based eating, psychological distress, and binge eating contribute causally to BMI, as opposed to the converse. Given this model, both BMI and recruitment groups status represents colliders, which means that covarying for them would introduce potential bias in parameter estimates by opening previously blocked causal paths from exposure to outcome [31], and thus these were not included as adjusted variables in our model.

Percentile bootstrapping (5000 samples) was utilised to calculate the standard errors and 95% CIs for indirect effects. Mediation was considered significant if the 95% CIs excluded zero. Normality of residuals was assessed through examination of histograms of standardised residuals. Homoscedasticity was assessed through examination of standardised residuals against predicted values. Cook’s distance values (<1) were used to assess any influential cases and variance inflation factors (VIFs) were used to assess multicollinearity.

## 3. Results

### 3.1. Descriptive Statistics

Table 1 presents the descriptive statistics and bivariate correlations for study variables. Participants were predominantly female (*n* = 132, 75%), and on average, middle aged (*M* = 43.27, *SD* = 11.74 years). Correlations between variables were generally weak or not statistically significant. However, there was a significant strong correlation between binge eating and reward-based eating (*r *= 0.75, *p* < 0.001), and a moderate correlation between binge eating and general distress (*r *= 0.41, *p* < 0.001).

### 3.2. Mediation Analyses

**Figure 1:**
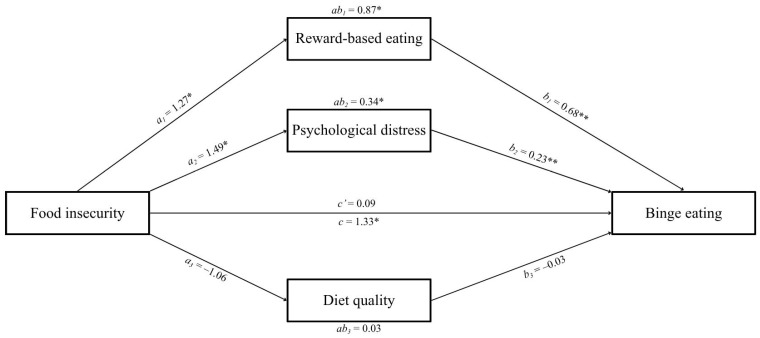
Results of the mediation analysis of food insecurity on binge eating. Note. * *p* < 0.05, ** *p* < 0.001. Coefficient values are unstandardised. Age, gender, and education were included as covariates in the mediation analysis but omitted for simplicity.

Assumption testing indicated no issues with normality of residuals, heteroscedasticity, multicollinearity or influential cases. Figure 1 shows the mediation model, with significant paths highlighted. Table 2 presents the mediation results while controlling for covariates (age, gender, education).

The total effect (*c* path) of food insecurity on binge eating was statistically significant. However, when accounting for the mediators of reward-based eating, psychological distress, and diet quality, the direct effect (*c*′ path) of food insecurity was not statistically significant. Results therefore suggest that the effect of food insecurity on binge eating is consistent with mediation through the included mechanisms. The indirect effects of food insecurity on binge eating were significant for both reward-based eating and psychological distress, but not diet quality, suggesting that reward-based eating and psychological distress, but not diet quality, mediated the association.

## 4. Discussion

We examined reward-based eating, psychological distress, and diet quality as potential mechanisms underpinning the association between food insecurity and binge eating. Our results demonstrated that psychological distress and reward-based eating, but not diet quality, mediated the association. The results provide support for the theory that food insecurity leads to a resource-imposed cycle of restriction, making individuals susceptible to binge eating via reward-based sensitivity. More specifically, our results suggest that food insecurity and the associated sense of deprivation may lead to increased responsiveness to food cues and preoccupation with food, resulting in increased risk of binge eating. Our results also provide support for the affect regulation model, whereby food insecurity and the associated sense of psychological distress results in a greater vulnerability to binge eating as an attempt to cope with negative emotional experiences [11]. While people experiencing food insecurity report purchasing energy-dense and processed foods due to their affordability [16], our results do not support diet quality as a key factor underlying food insecurity and binge eating.

In the current study, greater food insecurity was associated with increased reward-based eating and binge eating. To our knowledge, this was the first study to specifically explore reward-based eating as a mediator between food insecurity and binge eating. However, our findings are consistent with the proposed feast/famine model of binge eating associated with an externally imposed restriction of food insecurity. Our results suggest that limited access to food may increase preoccupation with food and loss of control over eating when food becomes available. When food is scarce or unpredictable, food may become more rewarding and there may be increased responsiveness towards such cues. This supports previous research where food insecurity was associated with greater food responsiveness and qualitative reports of a hedonic element of food consumption when desirable foods become available to those experiencing food insecurity [9,17]. Findings suggest that heightened appetitive reward processes may be one path which food insecurity confers risk of binge eating.

Our finding that psychological distress (comprising experiences of depression, anxiety, and stress) mediates the association between food insecurity and binge eating is also consistent with previous research. Stress and depression have independently been found to mediate associations between food insecurity and binge eating and related disorders [17,32]. This is consistent with qualitative reports where individuals with both food insecurity and binge eating unanimously reported that binge eating occurred in response to negative emotional experiences, most commonly stress [16]. Negative emotional experiences are a well-established precipitant of binge eating, beyond those who experience food insecurity [11]; however, this association may be particularly pertinent for individuals who may also have increased distress relating to uncertainty about accessing food due to financial pressures and low mood associated with food insecurity [16].

Previous research has found that greater food insecurity is associated with poorer diet quality [19]. Given the increased affordability and availability of foods with less nutritional value, which also tend to be foods consumed during binge eating, we expected diet quality to partially explain the association between food insecurity and binge eating. However, in contrast to our hypothesis, this pathway was not significant in the current study. Qualitative studies have highlighted that individuals experiencing food insecurity are more likely to purchase less nutritious, “binge promoting” foods when financial resources allow, resulting in additional episodes of binge eating [16]. Given we used a summary score of diet quality, it may be that the measurement index used in the current study was insufficiently sensitive to detect an effect. Alternatively, while binge eating often involves energy-dense and less nutritious foods, which happen to be more affordable and accessible, it may be the case that psychological factors play a stronger role in explaining the association.

Our findings have several implications. First, the association between food insecurity and binge eating highlights food security as a social target that should be considered as a factor in treatment approaches to binge eating. While the association was explained by key psychological factors, food insecurity may contribute and potentially exacerbate binge eating experiences for those who are vulnerable. Directly targeting food insecurity through relevant social policies is therefore indicated. Given regular eating is a key factor in recovery from binge eating [33], it may be poignant to consider how social policies could enable regular, stable, and increased access to food in food-insecure households.

Our findings that both reward-based eating and psychological distress were relevant in explaining the association between food insecurity and binge eating highlight the relevance of pathways involving both positive and negative emotional experiences. Beyond targeting the initial availability of food (i.e., food insecurity directly), treatment implications may include emotional regulation strategies or third-wave therapies to improve awareness of motivational and emotional states that may fuel overconsumption when food becomes available. Emotion regulation and broader mindfulness strategies could support management of both reward elements (such as urges) as well as negative emotional states and could therefore be important to consider in psychological treatment approaches to binge eating amongst those with co-occurring food insecurity.

This study involved a novel investigation into factors that may underpin the association between food insecurity and binge eating. However, the findings should be considered in light of several study limitations: First, the cross-sectional nature of the study means that strong causal claims are limited because the temporal ordering required to support causal inference cannot be established. Moreover, the original case–control design limits the generalisability of the findings. Whilst our study benefited from including groups that were deliberately oversampled from the population (i.e., healthy control with no psychopathology, higher weight groups with and without binge eating disorders) because it can help to increase statistical power to detect subtle effects that would be difficult to identify in the general population, it does mean that the effect sizes observed in the current study may be either attenuated or exaggerated when estimated in a population-representative sample.

As is common with research into eating disorders [34], our sample was largely female, with no participants identifying as gender-diverse. We also did not measure income or financial strain, which may have been an additional confounding factor. Future research should therefore aim to utilise longitudinal designs in a large and diverse sample of the general population to further investigate the directionality of associations between identified variables. Using more precise methods to investigate whether food insecurity and binge eating is explained by increased access and consumption of less nutritious and more palatable foods is also warranted.

## 5. Conclusions

This study provided novel evidence that the association between food insecurity and binge eating is underpinned by psychological factors including psychological distress and reward-based eating. Our findings highlight that both social and psychological intervention may be relevant in the treatment of binge eating in this population. Future studies are needed to replicate these findings in more diverse populations, explore associations longitudinally and investigate what interventions are most effective for this group of individuals.

## Figures and Tables

**Table 1:** Descriptive statistics and correlations for study variables (*N* = 176).

Variable	*M* (*SD*)	1	2	3	4	5	6	7	8
1. Gender ^a^	132 (75) ^b^	-							
2. Age	43.27 (11.74)	0.19 *	-						
3. Education	15.86 (2.80)	0.09	0.09	-					
4. Food Security	0.68 (1.35)	−0.16 *	−0.07	−0.13	-				
5. Reward-based Eating	28.02 (9.83)	0.06	0.10	−0.10	0.17 *	-			
6. Psychological Distress	12.82 (9.29)	−0.00	−0.02	−0.06	0.22 **	0.29 **	-		
7. Diet Quality	33.34 (10.39)	0.14	0.18 *	−0.02	−0.16 *	−0.00	−0.02	-	
8. Binge eating	15.03 (9.85)	0.12	0.10	−0.08	0.16 *	0.75 **	0.41 **	−0.03	-

*Note.* ^a^ 0 = Male, 1 = Female. ^b^ Data is *n* (%) of females. * *p* < 0.05. ** *p* < 0.01.

**Table 2:** Mediation effects of mechanisms between food insecurity and binge eating.

Variable	Effect	*b*	SE	95% CI	*p*
RED-13	*a1*	1.27	0.55	0.19, 2.36	0.022
	*b1*	0.68	0.05	0.58, 0.78	<0.001
	*ab1*	0.87	0.32	0.28, 1.54	-
DASS-21	*a2*	1.49	0.52	0.46, 2.52	0.005
	*b2*	0.23	0.05	0.12, 0.33	<0.001
	*ab2*	0.34	0.16	0.08, 0.69	-
AES	*a3*	−1.06	0.58	−2.21, 0.08	0.069
	*b3*	−0.03	0.05	−0.13, 0.06	0.494
	*ab3*	0.03	0.06	−0.07, 0.16	-
HFSSM-6	*c*	1.33	0.55	0.24, 2.42	0.017
	*c*′	0.09	0.37	−0.64, 0.83	0.800

*Note.* SEs and 95% CIs are bootstrapped estimates for indirect effects (ab1, ab2, ab3). For indirect effects, the mediation is statistically significant if the 95% CI does not include zero. Age, gender, and education were included as covariates in the mediation analysis but omitted for simplicity.

## Data Availability

The dataset generated and/or analysed during the current study is not publicly available but may be available from the corresponding author upon reasonable request.

## Supplementary Materials

The following supporting information can be downloaded at: https://www.mdpi.com/article/10.3390/nu18132126/s1, Figure S1: Directed Acyclic Graph.

## References

[1] Hazzard V.M., Loth K.A., Hooper L., Becker C.B. (2020). Food Insecurity and Eating Disorders: A Review of Emerging Evidence. Curr. Psychiatry Rep..

[2] Peebles R., Wilson J.L., Litt I.F., Hardy K.K., Lock J.D., Mann J.R., Borzekowski D.L.G. (2012). Disordered Eating in a Digital Age: Eating Behaviors, Health, and Quality of Life in Users of Websites with pro-Eating Disorder Content. J. Med. Internet Res..

[3] Food Insecurity. 2023|Australian Bureau of Statistics. https://www.abs.gov.au/statistics/health/food-and-nutrition/food-insecurity/latest-release.

[4] World Food Programme (WFP) (2025). WFP 2026 Global Outlook. Hunger and Hope: Innovative Solutions to Address Food Insecurity.

[5] Norris K., Pitts S.J., Reis H., Haynes-Maslow L. (2023). A Systematic Literature Review of Nutrition Interventions Implemented to Address Food Insecurity as a Social Determinant of Health. Nutrients.

[6] Rasmusson G., Lydecker J.A., Coffino J.A., White M.A., Grilo C.M. (2019). Household Food Insecurity Is Associated with Binge-Eating Disorder and Obesity. Int. J. Eat. Disord..

[7] Abene J.A., Tong J., Minuk J., Lindenfeldar G., Chen Y., Chao A.M. (2023). Food Insecurity and Binge Eating: A Systematic Review and Meta-Analysis. Int. J. Eat. Disord..

[8] Barry M.R., Sonneville K.R., Leung C.W. (2021). Students with Food Insecurity Are More Likely to Screen Positive for an Eating Disorder at a Large, Public University in the Midwest. J. Acad. Nutr. Diet..

[9] Hazzard V.M., Rodriguez M., Neumark-Sztainer D., Berge J.M., Tanofsky-Kraff M., Laska M.N. (2026). “Fulfilling the Hunger”: A Qualitative Study to Understand the Etiology of Binge Eating in Adolescents Experiencing Food Insecurity. Appetite.

[10] Bodell L.P., Racine S.E. (2023). A Mechanistic Staging Model of Reward Processing Alterations in Individuals with Binge-Type Eating Disorders. Int. J. Eat. Disord..

[11] Haedt-Matt A.A., Keel P.K. (2011). Revisiting the Affect Regulation Model of Binge Eating: A Meta-Analysis of Studies Using Ecological Momentary Assessment. Psychol. Bull..

[12] Coffino J.A., Spoor S.P., Drach R.D., Hormes J.M. (2021). Food Insecurity among Graduate Students: Prevalence and Association with Depression, Anxiety and Stress. Public Health Nutr..

[13] Pourmotabbed A., Moradi S., Babaei A., Ghavami A., Mohammadi H., Jalili C., Symonds M.E., Miraghajani M. (2020). Food Insecurity and Mental Health: A Systematic Review and Meta-Analysis. Public Health Nutr..

[14] Jandaghian-Bidgoli M., Kazemian E., Shaterian N., Abdi F. (2024). Focusing Attention on the Important Association between Food Insecurity and Psychological Distress: A Systematic Review and Meta-Analysis. BMC Nutr..

[15] Arenas D.J., Thomas A., Wang J., DeLisser H.M. (2019). A Systematic Review and Meta-Analysis of Depression, Anxiety, and Sleep Disorders in US Adults with Food Insecurity. J. Gen. Intern. Med..

[16] Green E.A., Schneider K.L., Chang A., Feinstein B.A., Rooper I.R., Wildes J.E., Graham A.K. (2025). Exploring Drivers of Binge Eating in Individuals With Food Insecurity and Recurrent Binge Eating: A Qualitative Analysis. Int. J. Eat. Disord..

[17] Kosmas J.A., Wildes J.E., Graham A.K., O’Connor S.M. (2023). The Role of Stress in the Association among Food Insecurity, Eating Disorder Pathology, and Binge Eating-Related Appetitive Traits. Eat. Behav..

[18] Worth I.A., Fazzino T.L., Gearhardt A.N. (2026). A 50 Year Review of Hyperpalatable Foods Consumed in Binge-Eating Episodes. Rev. Endocr. Metab. Disord..

[19] Kent K., Schumacher T., Kocar S., Seivwright A., Visentin D., Collins C.E., Lester L. (2024). Increasing Food Insecurity Severity Is Associated with Lower Diet Quality. Public Health Nutr..

[20] van der Velde L.A., Zitman F.M., Mackenbach J.D., Numans M.E., Kiefte-de Jong J.C. (2022). The Interplay between Fast-Food Outlet Exposure, Household Food Insecurity and Diet Quality in Disadvantaged Districts. Public Health Nutr..

[21] Rummo P.E., Guilkey D.K., Ng S.W., Popkin B.M., Evenson K.R., Gordon-Larsen P. (2017). Beyond Supermarkets: Food Outlet Location Selection in Four U.S. Cities Over Time. Am. J. Prev. Med..

[22] Colton E., Agosta C., Carey H., Giddens E., Noy B., Flanagan K., Tsyrlin L., Wilson K.-E., Pacheco L.B., Mahlberg J. (2026). Profiling Decision-Making Mechanisms in Binge Eating Disorder. J. Eat. Disord..

[23] First M.B., Williams J.B.W., Karg R.S., Spitzer R.L. (2015). Structured Clinical Interview for DSM-5—Research Version (SCID-5 for DSM-5, Research Version; SCID-5-RV).

[24] U.S. Department of Agriculture, Economic Research Service (2012). U.S. Household Food Security Survey Module: Six-Item Short Form.

[25] Mason A.E., Vainik U., Acree M., Tomiyama A.J., Dagher A., Epel E.S., Hecht F.M. (2017). Improving Assessment of the Spectrum of Reward-Related Eating: The RED-13. Front. Psychol..

[26] Henry J.D., Crawford J.R. (2005). The Short-Form Version of the Depression Anxiety Stress Scales (DASS-21): Construct Validity and Normative Data in a Large Non-Clinical Sample. Br. J. Clin. Psychol..

[27] Lovibond P.F., Lovibond S.H. (1995). The Structure of Negative Emotional States: Comparison of the Depression Anxiety Stress Scales (DASS) with the Beck Depression and Anxiety Inventories. Behav. Res. Ther..

[28] Collins C.E., Boggess M.M., Watson J.F., Guest M., Duncanson K., Pezdirc K., Rollo M., Hutchesson M.J., Burrows T.L. (2014). Reproducibility and Comparative Validity of a Food Frequency Questionnaire for Australian Adults. Clin. Nutr..

[29] Gormally J., Black S., Daston S., Rardin D. (1982). The Assessment of Binge Eating Severity among Obese Persons. Addict. Behav..

[30] Tennant P.W.G., Murray E.J., Arnold K.F., Berrie L., Fox M.P., Gadd S.C., Harrison W.J., Keeble C., Ranker L.R., Textor J. (2021). Use of Directed Acyclic Graphs (DAGs) to Identify Confounders in Applied Health Research: Review and Recommendations. Int. J. Epidemiol..

[31] VanderWeele T.J. (2019). Principles of Confounder Selection. Eur. J. Epidemiol..

[32] Zickgraf H.F., Stefano E., Price J., Veldheer S., Rogers A., Rigby A. (2019). The Relationship between Food Insecurity and Binge and Night Eating Symptoms in Prebariatric Surgery Patients Is Mediated by Depressive Symptoms. Surg. Obes. Relat. Dis..

[33] Zendegui E.A., West J.A., Zandberg L.J. (2014). Binge Eating Frequency and Regular Eating Adherence: The Role of Eating Pattern in Cognitive Behavioral Guided Self-Help. Eat. Behav..

[34] Burnette C.B., Luzier J.L., Weisenmuller C.M., Boutté R.L. (2022). A Systematic Review of Sociodemographic Reporting and Representation in Eating Disorder Psychotherapy Treatment Trials in the United States. Int. J. Eat. Disord..

