# Supplementary figures and images for: Food Insecurity and Binge Eating: Exploring Reward-Based Eating, Psychological Distress, and Diet Quality as Underlying Mechanisms
Source: Nutrients. 2026 Jul 1;18(13):2126. doi: 10.3390/nu18132126 (PMC13363440; doi:10.3390/nu18132126)

**Supplementary Figure S1. Directed Acyclic Graph**

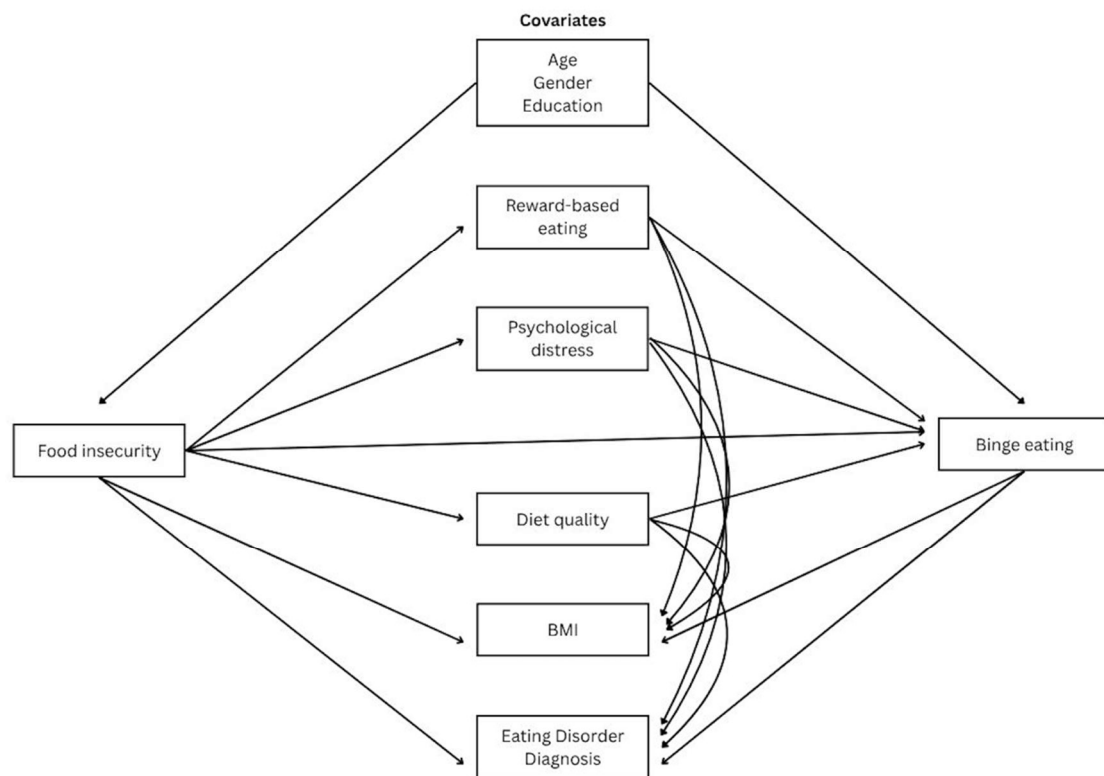

Note:

Arrows indicate causal pathways.

Supplement: Supplementary file 1 [file nutrients-18-02126-s001.zip › nutrients-4347272-supplementary.pdf]
